# Calreticulin and heat shock protein 70 expression, immune infiltration, and prognostic stratification in endometrial cancer: a retrospective cohort study

**DOI:** 10.3389/fonc.2026.1884530

**Published:** 2026-08-17

**Authors:** Xueqin Xiang, Xiaoqing Yin, Wenbin Zhan, Quanzhe Cui

**Affiliations:** 1 Department of Obstetrics and Gynecology, The Third People’s Hospital of Chengdu, Chengdu, China; 2 Department of Pathology, The Third People’s Hospital of Chengdu, Chengdu, China

**Keywords:** calreticulin, endometrial cancer, HSP70, immune infiltration, immunohistochemistry, prognosis, recurrence-free survival, risk stratification

## Abstract

**Background:**

Accessible biomarkers are needed to complement conventional clinicopathological risk assessment in endometrial cancer. Calreticulin (CALR) and heat shock protein 70 (HSP70) are stress-related proteins involved in immunogenic cell death and tumor–immune interactions, but their clinicopathological and prognostic relevance remains unclear.

**Methods:**

This retrospective cohort included 286 surgically treated patients who underwent surgery between January 2016 and December 2023, with administrative follow-up through December 31, 2025. CALR and HSP70 expression was assessed by immunohistochemistry and classified as low or high using prespecified semiquantitative thresholds. CD8+ and CD68+ cell densities were evaluated as immune infiltration markers. Associations with adverse pathological features were examined using logistic regression with false-discovery rate correction. Recurrence-free survival (RFS), defined as recurrence or death, and overall survival (OS) were analyzed using Kaplan-Meier curves and Cox regression. Clinical and integrated models were compared for 3-year recurrence prediction with bootstrap validation.

**Results:**

High CALR and high HSP70 expression were independently associated with grade 3 disease, deep myometrial invasion, lymphovascular space invasion, lymph node metastasis, advanced clinicopathological stage, and a secondary adverse pathology summary endpoint after false-discovery rate correction. High expression of either marker was associated with lower CD8+ and higher CD68+ density. In multivariable RFS models including adjuvant treatment, HSP70-high expression remained associated with poorer RFS (adjusted HR 1.85, 95% CI 1.23–2.79; P = 0.003), whereas CALR-high expression did not. The CALR-high/HSP70-high phenotype was also associated with poorer RFS (adjusted HR 2.30, 95% CI 1.36–3.88; P = 0.002). OS models were exploratory because of limited deaths, although associations for HSP70-high expression and the double-high phenotype were directionally consistent. For 3-year recurrence prediction, the integrated model showed a small apparent AUC increase over the clinical model (0.779 vs. 0.763; DeLong P = 0.350), which was not retained after bootstrap optimism correction (0.717 vs. 0.720).

**Conclusion:**

CALR and HSP70 expression were associated with aggressive pathology and an immune profile characterized by reduced CD8+ and increased CD68+ infiltration. HSP70 and the CALR-high/HSP70-high phenotype showed the most consistent RFS associations, whereas OS and prediction-model findings require cautious interpretation.

## 1 Introduction

Endometrial cancer is one of the most common gynecologic malignancies worldwide, and its burden continues to increase in many regions in parallel with population aging and the rising prevalence of obesity-related metabolic disorders (1–3). Although a substantial proportion of patients are diagnosed at an early stage and achieve favorable outcomes after primary surgery, clinical behavior remains heterogeneous. In routine practice, some tumors with apparently similar clinicopathological features follow markedly different courses, suggesting that conventional prognostic variables alone do not fully capture the underlying biological diversity of the disease (4–6).

Over the past decade, the management of endometrial cancer has moved toward more refined risk stratification. The integrated genomic analysis by The Cancer Genome Atlas (TCGA) established four biologically and clinically distinct molecular subgroups, and this framework has subsequently influenced modern staging and guideline recommendations (5–7). At the same time, the implementation of full molecular classification is still uneven across institutions, and histology, grade, depth of myometrial invasion, lymphovascular space invasion (LVSI), nodal status, and other pathology-based variables remain central to decision-making in many settings (4, 7). This creates a practical need for additional, low-cost biomarkers that can be assessed on routine tissue sections and that may complement standard clinicopathological evaluation.

Another layer of heterogeneity in endometrial cancer lies in the tumor immune microenvironment. Increasing evidence suggests that immune cell composition is not merely descriptive, but clinically relevant. Higher intratumoral CD8+ T-cell infiltration has been associated with more favorable outcomes, whereas macrophage-rich microenvironments have been linked to invasive behavior and poorer prognosis (8–10). These observations support the view that the local immune context may influence not only tumor progression but also the prognostic meaning of pathology-based biomarkers. However, the interaction between stress-related tumor markers and immune infiltration patterns in endometrial cancer remains insufficiently defined.

CD8-positive cells were evaluated as a marker of cytotoxic T-cell infiltration, whereas CD68-positive cells were evaluated as a broad macrophage marker, including the tumor-associated macrophage compartment. Considering CD8 and CD68 together provides a simple pathology-based view of the balance between cytotoxic lymphoid infiltration and macrophage-enriched myeloid inflammation, which is relevant when interpreting CALR/HSP70-related tumor stress and immune remodeling.

Calreticulin (CALR) and heat shock protein 70 (HSP70) are of particular interest in this setting. Both are stress-responsive chaperone proteins that have been implicated in immunogenic cell death and damage-associated molecular pattern signaling (11). Surface-exposed CALR can act as a pro-phagocytic signal, while HSP70 has complex intracellular and extracellular functions that may influence antigen presentation, cellular stress tolerance, and tumor-immune interactions (12, 13). At the same time, their role in cancer is context dependent. Tumor-associated overexpression detected by immunohistochemistry does not necessarily indicate effective antitumor immunity; in some settings, it may instead reflect adaptive stress signaling, resistance to cell death, or more aggressive tumor biology (14, 15).

In endometrial cancer, the available literature on these markers remains limited and somewhat inconsistent. A prior study suggested that low CALR expression was associated with aggressive progression and poor prognosis in endometrial cancer (16), whereas older studies reported that HSP70 expression correlated with unfavorable clinicopathological features and was linked to poorer outcomes (17, 18). These findings are suggestive, but they leave several questions unresolved. In particular, the clinicopathological and prognostic implications of evaluating CALR and HSP70 together, rather than separately, have not been well characterized. Likewise, their relationship with CD8+ and CD68+ immune infiltration in endometrial cancer has not been systematically addressed in a routine pathology-based cohort.

Against this background, we hypothesized that high tumor-cell CALR and HSP70 expression would identify a stress-adapted tumor phenotype associated with adverse pathology, an immune-infiltration pattern characterized by lower CD8+ and higher CD68+ cell density, and poorer clinical outcomes. The present study therefore aimed to assess CALR and HSP70 expression in surgically treated endometrial cancer, examine their associations with aggressive clinicopathological features and immune infiltration, evaluate their individual and combined prognostic value for RFS and OS, and explore whether integrating these biomarkers with immune variables could improve recurrence risk stratification beyond conventional clinical factors.

## 2 Materials and methods

### 2.1 Study design and patient selection

This retrospective cohort study included patients with endometrial cancer who underwent surgical treatment at The Third People’s Hospital of Chengdu between January 2016 and December 2023. Consecutive cases were screened through the institutional medical record system, and the administrative data-lock date was December 31, 2025. Patients were eligible if they had pathologically confirmed primary endometrial cancer, underwent radical surgery, had available formalin-fixed paraffin-embedded tumor tissue for immunohistochemical assessment, and had complete clinicopathological and follow-up data. Patients were excluded if they had non-primary or non-endometrial malignancies, did not undergo radical surgery, lacked CALR or HSP70 IHC data, had incomplete key clinicopathological information, or had inadequate follow-up for outcome assessment. The final analytic cohort was defined according to these prespecified criteria.

This study was conducted in accordance with the Declaration of Helsinki. This study was approved by the institutional review board of The Third People’s Hospital of Chengdu (approval No. cdth023119). Given the retrospective nature of the study and the use of de-identified data, the requirement for written informed consent was waived.

### 2.2 Clinicopathological data collection

Clinicopathological variables were extracted from the medical record system and pathology reports. The collected variables included age at surgery, body mass index (BMI), menopausal status, hypertension, diabetes, preoperative CA125 level, histological subtype, histological grade, tumor size, depth of myometrial invasion, cervical stromal involvement, lymphovascular space invasion (LVSI), serosal/adnexal involvement, lymph node metastasis, FIGO stage, adjuvant treatment, recurrence status, RFS, OS, and vital status at last follow-up.

Histological subtype was categorized as endometrioid, serous, clear cell, or mixed carcinoma. Histological grade was recorded as grade 1, 2, or 3. Myometrial invasion was classified as <50% or ≥50%. FIGO stage was recorded from available clinicopathological staging information in the pathology reports. Because molecular subtype information and exact IA/IB substaging were not available for all cases, the present study did not apply full FIGO 2023 molecularly integrated staging. Advanced clinicopathological stage was defined as stage III-IV (19). As a secondary descriptive summary, an adverse pathology summary endpoint was defined as the presence of at least one of the following adverse features: grade 3 disease, deep myometrial invasion (≥50%), LVSI, lymph node metastasis, or advanced clinicopathological stage.

### 2.3 Immunohistochemistry and biomarker assessment

Immunohistochemistry was performed on 4-μm-thick sections cut from formalin-fixed, paraffin-embedded tumor blocks. Sections were mounted on charged glass slides, baked at 60 °C for 1 h, deparaffinized in xylene, and rehydrated through graded ethanol. Heat-induced epitope retrieval was performed in EDTA buffer (pH 9.0) at 95-98 °C for 20 min, followed by cooling at room temperature for 20 min. Endogenous peroxidase activity was blocked with 3% hydrogen peroxide for 10 min. Staining was performed manually using a polymer-based horseradish peroxidase detection system, with 3,3’-diaminobenzidine as the chromogen and hematoxylin counterstaining. The primary antibodies were as follows: anti-CALR rabbit monoclonal antibody, clone EPR3924 (Abcam, Cambridge, UK; ab92516; 1:250); anti-HSP70 mouse monoclonal antibody, clone C92F3A-5 (Abcam; ab47455; 1:1,000); anti-CD8 mouse monoclonal antibody, clone C8/144B (Dako/Agilent Technologies, Santa Clara, CA, USA; M7103; 1:100); and anti-CD68 mouse monoclonal antibody, clone KP1 (Dako/Agilent Technologies; M0814; 1:100). Primary antibodies were incubated overnight at 4 °C, followed by incubation with the secondary polymer reagent according to the manufacturer’s protocol. Positive controls were included in each staining run: normal human kidney tissue for CALR because of its reproducible endoplasmic-reticulum-associated staining, previously confirmed HSP70-positive carcinoma tissue for HSP70, and human tonsil tissue for CD8 and CD68. Negative controls were prepared by replacing the primary antibody with phosphate-buffered saline or species- and isotype-matched nonimmune IgG at the corresponding concentration. Representative tumor sections were reviewed by pathologists who were blinded to clinicopathological and outcome data.

CALR and HSP70 expression were evaluated using a semiquantitative scoring approach based on staining intensity and staining extent. Staining intensity was scored as 0, 1, 2, or 3, and staining extent was scored as 0, 1, 2, 3, or 4 according to the proportion of positive tumor cells. A total IHC score was calculated by multiplying intensity and extent scores, yielding a range from 0 to 12. For both CALR and HSP70, a total score of 0–5 was classified as low expression and a score of 6–12 as high expression (20).

The 0–5 versus 6–12 high/low threshold was prespecified before the outcome analyses and was retained for the primary analysis to maintain a simple pathology-facing classification. To address potential information loss from dichotomization, sensitivity analyses additionally modeled CALR and HSP70 total scores as continuous standardized variables and repeated the main analyses using alternative thresholds of 5 and 7. Inter-observer reproducibility of CALR and HSP70 scoring was evaluated using intraclass correlation coefficients for total scores and Cohen’s kappa for high/low classification.

For immune infiltration analysis, CD8- and CD68-positive cells were quantified manually in representative non-necrotic tumor areas on whole tumor sections and recorded as average cell density per high-power field (cells/HPF). Quantification focused on tumor-associated immune infiltrates; necrotic areas, crush artifact, and non-tumor tissue were excluded from counting. These variables were analyzed as continuous measures in the main analyses. For combined prognostic analyses, patients were further categorized into four groups according to CALR and HSP70 expression status: CALR-low/HSP70-low, CALR-high/HSP70-low, CALR-low/HSP70-high, and CALR-high/HSP70-high.

### 2.4 Follow-up and study endpoints

Patients were followed from the date of surgery until recurrence, death, last follow-up, or the administrative data-lock date of December 31, 2025, whichever came first. RFS was defined as the interval from surgery to the first documented recurrence or death, whichever occurred first; patients without recurrence or death were censored at last follow-up. A recurrence-only time-to-event sensitivity analysis was also performed. OS was defined as the interval from surgery to death from any cause or censoring at the last follow-up for surviving patients.

For model development, a binary 3-year recurrence endpoint was additionally generated. Patients who developed recurrence within 36 months after surgery were classified as having a 3-year recurrence event. Patients who remained recurrence-free for at least 36 months were classified as non-events. Patients who were recurrence-free but had less than 36 months of follow-up were not included in the 3-year recurrence model analysis.

### 2.5 Statistical analysis

All statistical analyses were performed using R software (R 4.5.3; R Foundation for Statistical Computing, Vienna, Austria). Continuous variables are presented as median with first and third quartiles (Q1, Q3), and categorical variables are presented as number with percentage. Baseline characteristics of the study cohort were summarized descriptively. Clinicopathological characteristics were compared between CALR-high and CALR-low groups and between HSP70-high and HSP70-low groups using the Wilcoxon rank-sum test for continuous variables and Fisher’s exact test for categorical variables.

To examine associations between biomarker expression and adverse clinicopathological features, univariable and multivariable logistic regression analyses were performed separately for CALR and HSP70. The prespecified pathological outcomes included grade 3 disease, deep myometrial invasion, lymphovascular space invasion (LVSI), lymph node metastasis, and advanced clinicopathological stage. The adverse pathology summary endpoint was analyzed as a secondary descriptive endpoint because advanced clinicopathological stage partly overlaps with component pathological features. In multivariable logistic regression, the models were adjusted for age, BMI, histology, and preoperative CA125. Benjamini-Hochberg false-discovery rate (FDR) adjustment was applied within prespecified pathology analyses.

Associations between CALR/HSP70 expression and immune infiltration were assessed by comparing CD8 and CD68 cell densities between high- and low-expression groups using nonparametric testing, and the distributions were visualized using violin plots with embedded boxplots.

Survival curves for RFS and OS were estimated using the Kaplan-Meier method and compared with the log-rank test. Numbers at risk were summarized at 0, 24, 48, 72, 96, and 120 months. Univariable and multivariable Cox proportional hazards regression models were used to assess the prognostic effects of CALR and HSP70 individually, as well as the combined CALR/HSP70 expression groups. In survival analyses of individual biomarkers, high expression was compared with low expression as the reference category. In combined-group analyses, the CALR-low/HSP70-low group served as the reference group. RFS models were adjusted for age, histology, grade, deep myometrial invasion, LVSI, lymph node metastasis, preoperative CA125, and adjuvant treatment, with sensitivity analyses excluding adjuvant treatment because it may partly reflect treatment indication. Given the limited number of deaths, OS multivariable analyses used reduced covariate sets and were interpreted as exploratory. Proportional-hazards assumptions were evaluated using Schoenfeld residuals, and stratified Cox sensitivity analyses were performed when appropriate.

Histological subtype was included as an adjustment covariate in the main multivariable models. Because non-endometrioid tumors were relatively uncommon, histology was not used as a primary four-level stratification variable. Instead, histology-oriented sensitivity analyses used an endometrioid versus non-endometrioid grouping, including full-cohort models with histology group and endometrioid-only sensitivity models.

To evaluate whether biomarker and immune variables improved recurrence risk prediction, multivariable logistic regression models were constructed for the binary 3-year recurrence endpoint. The clinical model included age, histology, grade, deep myometrial invasion, LVSI, lymph node metastasis, preoperative CA125, and adjuvant treatment. The integrated model included all variables in the clinical model plus CALR expression, HSP70 expression, CD8 density, and CD68 density. Variables were selected *a priori* according to clinical relevance and the study hypothesis, and no data-driven stepwise variable selection was used.

Internal validation of the prediction models was performed using bootstrap resampling. Briefly, 1,000 bootstrap samples with the same size as the analytic dataset were generated by sampling with replacement. In each bootstrap sample, the clinical and integrated models were refitted, and model performance was evaluated both in the bootstrap sample and in the original dataset. The average difference between these two estimates was used to quantify optimism, and optimism-corrected performance estimates were then derived for each model. Model discrimination was assessed using the area under the receiver operating characteristic curve (AUC). Apparent AUC 95% confidence intervals were estimated using the DeLong method, and optimism-corrected AUC 95% confidence intervals were estimated using bootstrap percentile intervals. The apparent AUCs of the two models were compared using the DeLong test for paired ROC curves, with the result interpreted together with the bootstrap-corrected performance estimates.

Model calibration was evaluated using calibration curves, calibration intercept and slope, and predicted versus observed recurrence probabilities across risk deciles. Overall prediction error was summarized using the Brier score. The integrated calibration index (ICI), median absolute calibration error (E50), and 90th percentile absolute calibration error (E90) were additionally calculated from risk-decile calibration summaries. Sensitivity and specificity were calculated at the optimal threshold determined by the Youden index. Decision curve analysis (DCA) was performed to compare the net clinical benefit of the clinical and integrated models across clinically relevant threshold probabilities.

All tests were two-sided. Nominal P values <0.05 were considered statistically significant, and FDR-adjusted P values were reported for families of related analyses where multiple testing was relevant. Biomarker reporting was reviewed against REMARK principles, including explicit endpoint definitions, prespecified covariates, cut-off sensitivity analyses, internal validation, and reproducibility assessment.

## 3 Results

### 3.1 Patient selection and baseline characteristics

**Figure 1:**
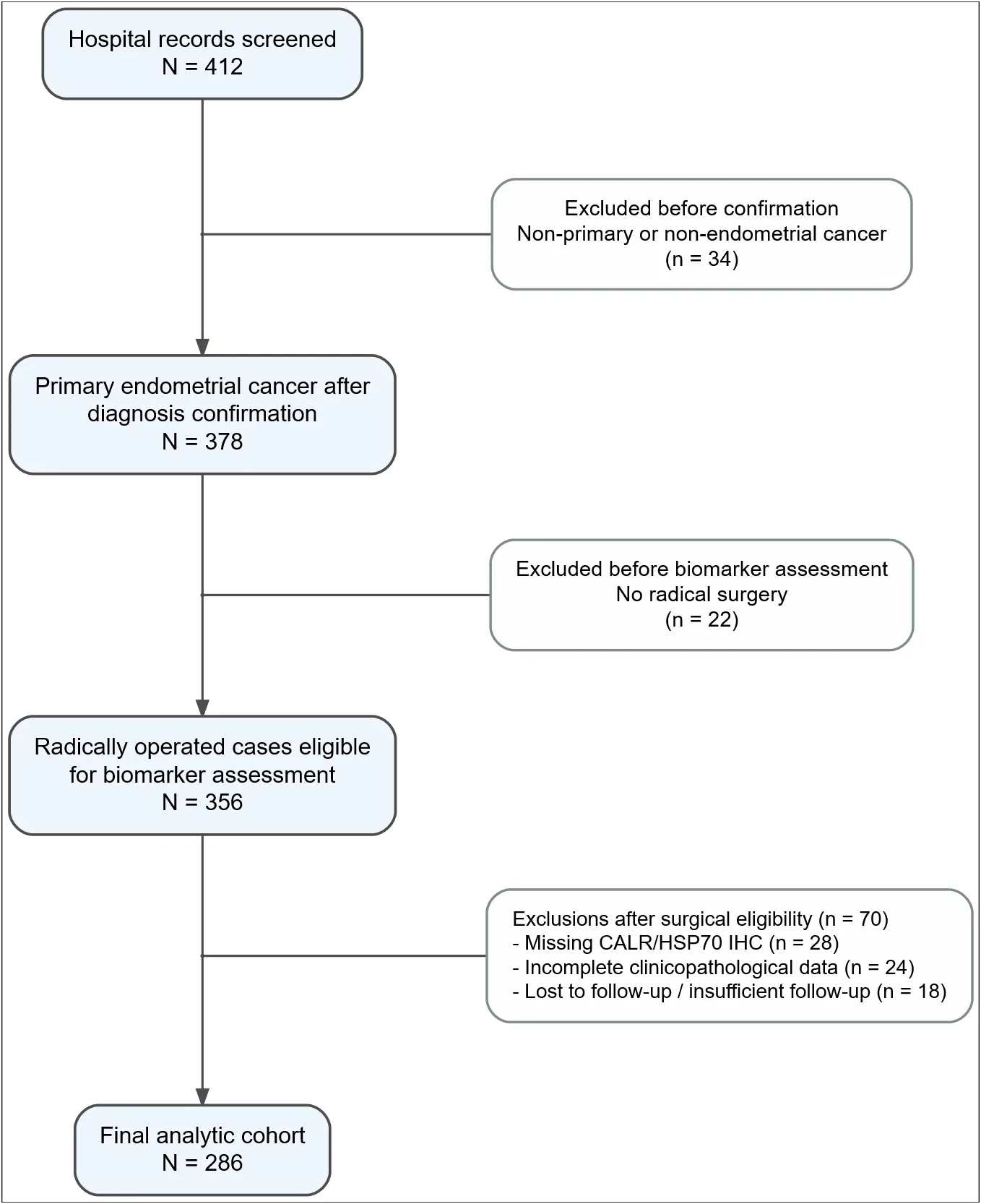
Flowchart of patient selection for the study cohort. Hospital records were screened to identify patients with primary endometrial cancer. After exclusion of non-primary or non-endometrial cancers (n = 34), cases without radical surgery (n = 22), missing CALR/HSP70 immunohistochemistry data (n = 28), incomplete clinicopathological data (n = 24), and loss to follow-up or insufficient follow-up (n = 18), a total of 286 patients were included in the final analytic cohort.

A total of 412 hospital records were initially screened. After exclusion of non-primary or non-endometrial cancers (n = 34), cases without radical surgery (n = 22), patients with missing CALR/HSP70 IHC data (n = 28), incomplete clinicopathological data (n = 24), and loss to follow-up or insufficient follow-up (n = 18), 286 patients were included in the final analytic cohort (**Figure 1**). Available screening-level characteristics of included and excluded records are summarized in **Supplementary Table S11**.

The baseline clinicopathological characteristics of the study population are summarized in **Table 1**. The median age was 57.0 years (Q1, Q3: 51.0, 63.0), and most patients were postmenopausal (90%). The median body mass index was 27.6 kg/m2 (Q1, Q3: 24.1, 31.0). Endometrioid histology was the predominant subtype (86%). With respect to tumor grade, 36% of cases were grade 1, 35% were grade 2, and 28% were grade 3. Deep myometrial invasion (≥50%) was observed in 31% of patients, cervical stromal involvement in 15%, LVSI in 33%, serosal/adnexal involvement in 10%, and lymph node metastasis in 16%. FIGO stage I disease accounted for 65% of cases, whereas 26.3% of patients had stage III-IV disease. The observed surgery dates ranged from January 4, 2016, to December 4, 2023, and the administrative data-lock date was December 31, 2025. During a median follow-up of 72.5 months (Q1, Q3: 45.8, 95.3; maximum, 119.9), recurrence occurred in 90 patients (31%), RFS events occurred in 126 patients (44%), and death occurred in 59 patients (21%).

**Table 1:** Baseline clinicopathological characteristics of the study cohort (N = 286).

Characteristic	N = 286
Age, years, Median (Q1, Q3)	57.0 (51.0, 63.0)
Age group, n (%)	
<50	52 (18%)
50–59	119 (42%)
60–69	90 (31%)
≥70	25 (8.7%)
BMI, kg/m^2^, Median (Q1, Q3)	27.6 (24.1, 31.0)
Menopausal status, n (%)	
Pre-menopause	28 (9.8%)
Post-menopause	258 (90%)
Hypertension, n (%)	64 (22%)
Diabetes, n (%)	50 (17%)
Preoperative CA125, U/mL, Median (Q1, Q3)	19.8 (13.7, 27.2)
Histology, n (%)	
Endometrioid	245 (86%)
Serous	24 (8.4%)
Clear cell	9 (3.1%)
Mixed	8 (2.8%)
Histological grade, n (%)	
G1	104 (36%)
G2	101 (35%)
G3	81 (28%)
Tumor size, cm, Median (Q1, Q3)	3.3 (2.5, 3.9)
Myometrial invasion, n (%)	
<50%	197 (69%)
≥50%	89 (31%)
Cervical stromal involvement, n (%)	42 (15%)
LVSI, n (%)	94 (33%)
Serosa/adnexa involvement, n (%)	30 (10%)
Lymph node metastasis, n (%)	46 (16%)
FIGO stage, n (%)	
I	187 (65%)
II	23 (8.0%)
III	58 (20%)
IV	18 (6.3%)
Adjuvant treatment, n (%)	
None	130 (45%)
Radiotherapy	58 (20%)
Chemotherapy	47 (16%)
Chemoradiotherapy	51 (18%)
Follow-up, months, Median (Q1, Q3)	72.5 (45.6, 95.3)
Recurrence, n (%)	90 (31%)
Death, n (%)	59 (21%)

Continuous variables are presented as median (Q1, Q3), and categorical variables are presented as n (%). Percentages may not sum to 100% due to rounding.

### 3.2 Clinicopathological features according to CALR and HSP70 expression

**Figure 2:**
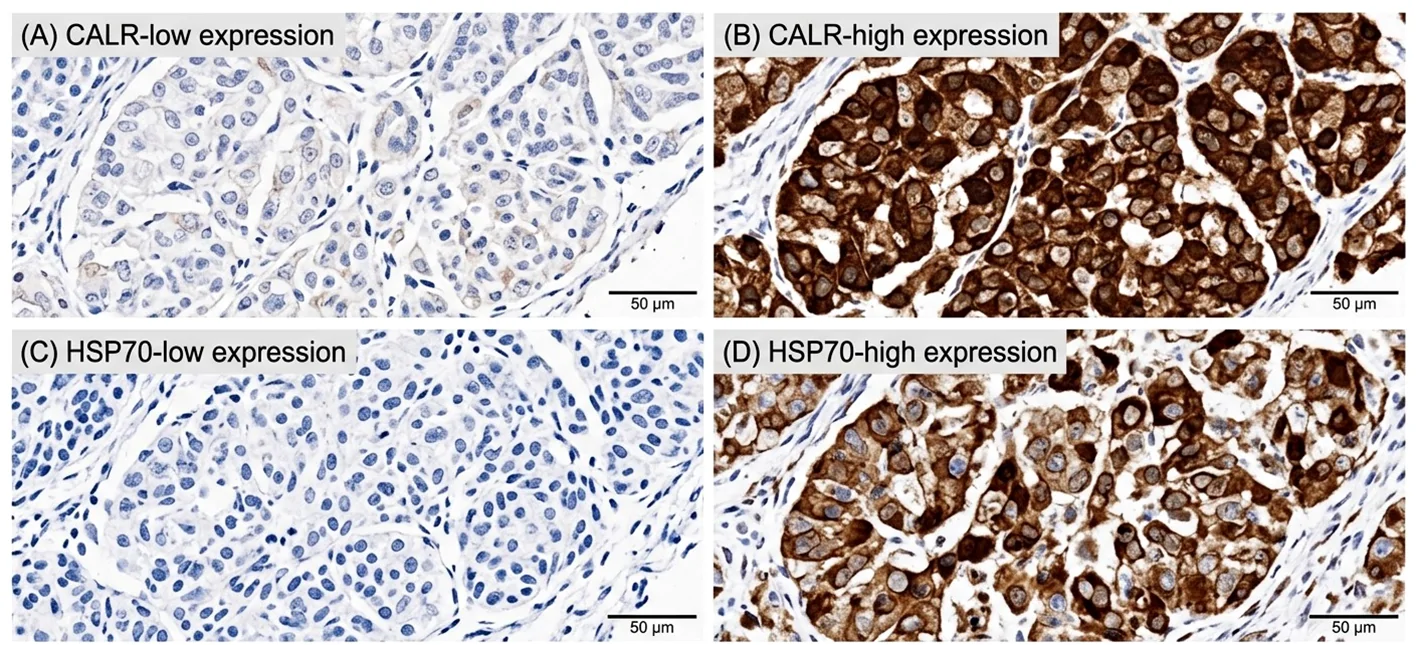
Representative immunohistochemical staining patterns of CALR and HSP70 in endometrial cancer tissues. **(A)** Low CALR expression, showing weak and limited immunoreactivity in tumor cells. **(B)** High CALR expression, showing strong and diffuse staining with prominent cytoplasmic and membranous reactivity. **(C)** Low HSP70 expression, showing weak or focal staining in tumor cells. **(D)** High HSP70 expression, showing strong and extensive predominantly cytoplasmic immunoreactivity. These panels illustrate the heterogeneous expression of CALR and HSP70 across tumor samples and support the semiquantitative classification of cases into low- and high-expression groups. Scale bars = 50 μm.

**Figure 2** shows representative low- and high-expression cases for CALR and HSP70 in endometrial cancer tissues. The visual contrast in staining intensity and extent supports the semiquantitative IHC assessment used to define biomarker expression groups, which were subsequently examined in relation to clinicopathological aggressiveness, immune infiltration, and patient outcomes. Clinicopathological comparisons according to CALR and HSP70 expression status are presented in **Supplementary Tables S1**, **S2**.

Inter-observer reproducibility of IHC assessment was high. Total-score ICCs were 0.949 (95% CI, 0.934-0.962) for CALR and 0.936 (95% CI, 0.921-0.950) for HSP70. High/low classification agreement was 90.9% for CALR and 91.3% for HSP70, with Cohen’s kappa values of 0.817 (95% CI, 0.747-0.881) and 0.825 (95% CI, 0.756-0.887), respectively (**Supplementary Table S6**). Sensitivity analyses using alternative IHC thresholds and continuous standardized total scores generally supported the direction of the main findings, although not every endpoint remained statistically significant under every alternative threshold (**Supplementary Table S7**).

Compared with the CALR-low group, patients in the CALR-high group more frequently showed grade 3 tumors, deep myometrial invasion, cervical stromal involvement, LVSI, serosal/adnexal involvement, lymph node metastasis, advanced clinicopathological stage, and receipt of adjuvant treatment (all P < 0.001). Preoperative CA125 levels also differed modestly between the two groups (P = 0.021), whereas age, body mass index, menopausal status, hypertension, diabetes, histologic subtype, and tumor size were not significantly different.

Similarly, the HSP70-high group showed significantly higher frequencies of grade 3 disease, deep myometrial invasion, cervical stromal involvement, LVSI, serosal/adnexal involvement, lymph node metastasis, advanced clinicopathological stage, and adjuvant treatment than the HSP70-low group. Histologic subtype also differed between the two HSP70 expression groups (P = 0.010), whereas age, body mass index, menopausal status, hypertension, diabetes, preoperative CA125, and tumor size were not significantly different.

### 3.3 Associations of CALR and HSP70 expression with immune infiltration

**Figure 3:**
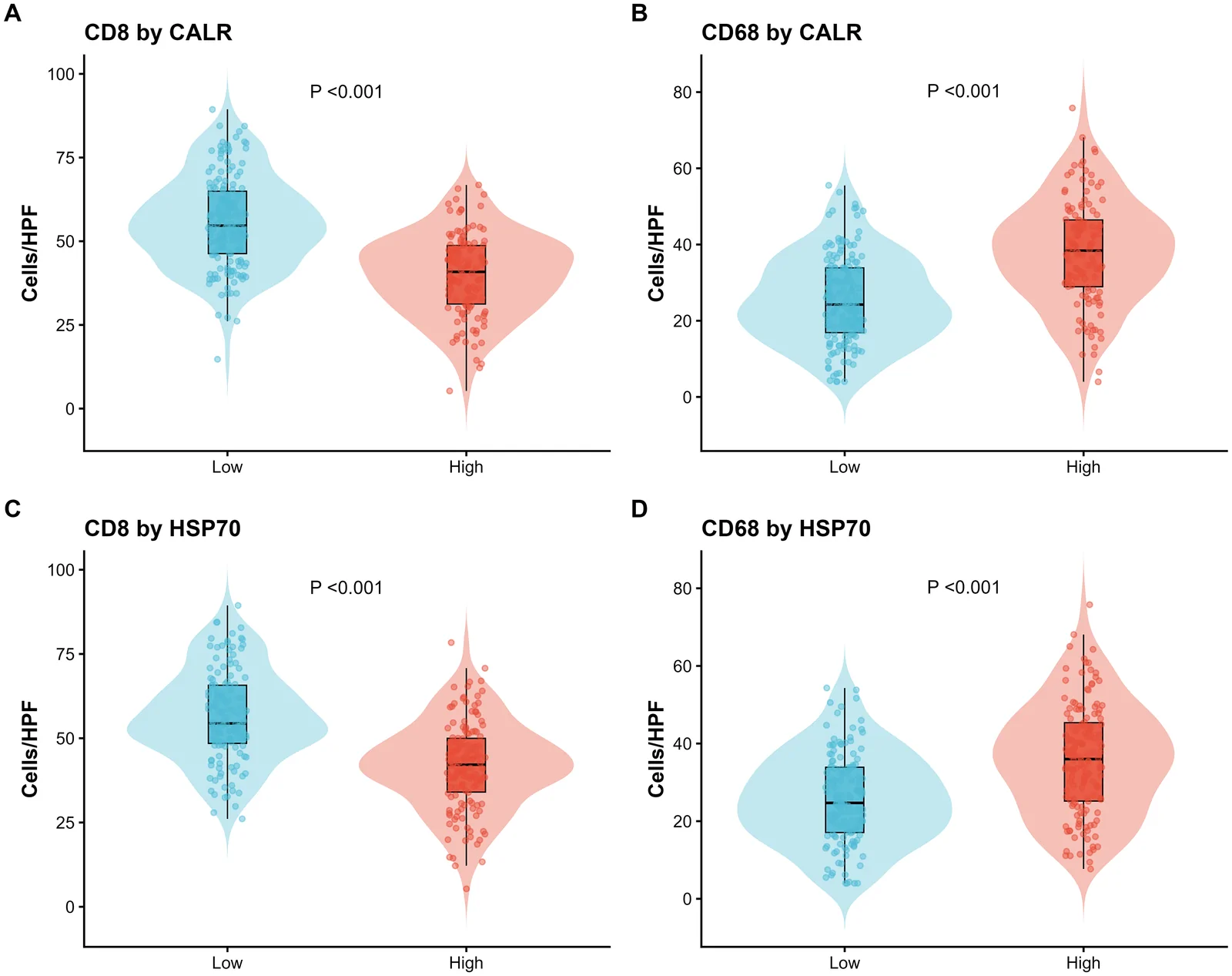
Associations of CALR and HSP70 expression with CD8+ and CD68+ immune cell infiltration in endometrial cancer. Violin plots with embedded boxplots and jittered data points show the distribution of immune cell densities according to CALR and HSP70 expression status. **(A)** CD8+ cell density according to CALR expression. **(B)** CD68+ cell density according to CALR expression. **(C)** CD8+ cell density according to HSP70 expression. **(D)** CD68+ cell density according to HSP70 expression. P values were calculated using the Wilcoxon rank-sum test.

The relationships between CALR/HSP70 expression and immune cell infiltration are shown in **Figure 3**. High CALR expression was associated with lower CD8+ cell density and higher CD68+ cell density than low CALR expression (both P < 0.001). A similar pattern was observed for HSP70, with HSP70-high tumors showing reduced CD8+ infiltration and increased CD68+ infiltration compared with HSP70-low tumors (both P < 0.001). In exploratory immune-pattern analyses, the double-high phenotype showed the lowest CD8/CD68 balance and a macrophage-skewed profile, whereas formal CALR-by-HSP70 interaction tests did not provide strong evidence of multiplicative interaction (**Supplementary Table S7**). These findings indicate that high CALR and HSP70 expression were associated with an immune microenvironment characterized by reduced cytotoxic T-cell infiltration and increased macrophage infiltration.

### 3.4 Associations of CALR and HSP70 with aggressive pathological features

Univariable logistic regression analyses are shown in **Supplementary Table S3**. In univariable analyses, both CALR and HSP70 high expression were significantly associated with grade 3 disease, deep myometrial invasion, LVSI, lymph node metastasis, advanced clinicopathological stage, and the adverse pathology summary endpoint. Most associations remained significant after FDR correction.

In multivariable logistic regression analyses (**Table 2**), CALR high expression remained independently associated with all evaluated adverse pathological features, including grade 3 disease (adjusted OR 3.44, 95% CI 1.91-6.39, P < 0.001), deep myometrial invasion (adjusted OR 2.73, 95% CI 1.63-4.62, P < 0.001), LVSI (adjusted OR 2.86, 95% CI 1.71-4.85, P < 0.001), lymph node metastasis (adjusted OR 4.08, 95% CI 2.07-8.49, P < 0.001), advanced clinicopathological stage (adjusted OR 4.37, 95% CI 2.49-7.88, P < 0.001), and the adverse pathology summary endpoint (adjusted OR 3.68, 95% CI 2.16-6.43, P < 0.001).

**Table 2:** Multivariable logistic regression analyses of CALR and HSP70 high expression for adverse pathological features in endometrial cancer.

Outcome	CALR, adjusted OR (95% CI)	*P* value	HSP70, adjusted OR (95% CI)	*P* value
Grade 3	3.44 (1.91-6.39)	<0.001	1.92 (1.08-3.47)	0.027
Deep myometrial invasion	2.73 (1.63-4.62)	<0.001	1.96 (1.17-3.33)	0.012
LVSI	2.86 (1.71-4.85)	<0.001	2.32 (1.38-3.94)	0.002
Lymph node metastasis	4.08 (2.07-8.49)	<0.001	4.73 (2.25-10.92)	<0.001
Advanced clinicopathological stage	4.37 (2.49-7.88)	<0.001	3.89 (2.17-7.23)	<0.001
Adverse pathology summary	3.68 (2.16-6.43)	<0.001	2.94 (1.77-4.95)	<0.001

High expression was compared with low expression as the reference category. Multivariable models were adjusted for age, BMI, histology, and preoperative CA125. Advanced clinicopathological stage was defined as stage III-IV based on available clinicopathological staging information. The adverse pathology summary endpoint was analyzed as a secondary descriptive endpoint. FDR-adjusted P values are presented in **Supplementary Table S7**.

HSP70 high expression also remained independently associated with grade 3 disease (adjusted OR 1.92, 95% CI 1.08-3.47, P = 0.027), deep myometrial invasion (adjusted OR 1.96, 95% CI 1.17-3.33, P = 0.012), LVSI (adjusted OR 2.32, 95% CI 1.38-3.94, P = 0.002), lymph node metastasis (adjusted OR 4.73, 95% CI 2.25-10.92, P < 0.001), advanced clinicopathological stage (adjusted OR 3.89, 95% CI 2.17-7.23, P < 0.001), and the adverse pathology summary endpoint (adjusted OR 2.94, 95% CI 1.77-4.95, P < 0.001). All primary pathology-feature associations remained significant after FDR correction (**Table 2**; **Supplementary Table S7**).

### 3.5 Survival analyses according to CALR and HSP70 expression

**Figure 4:**
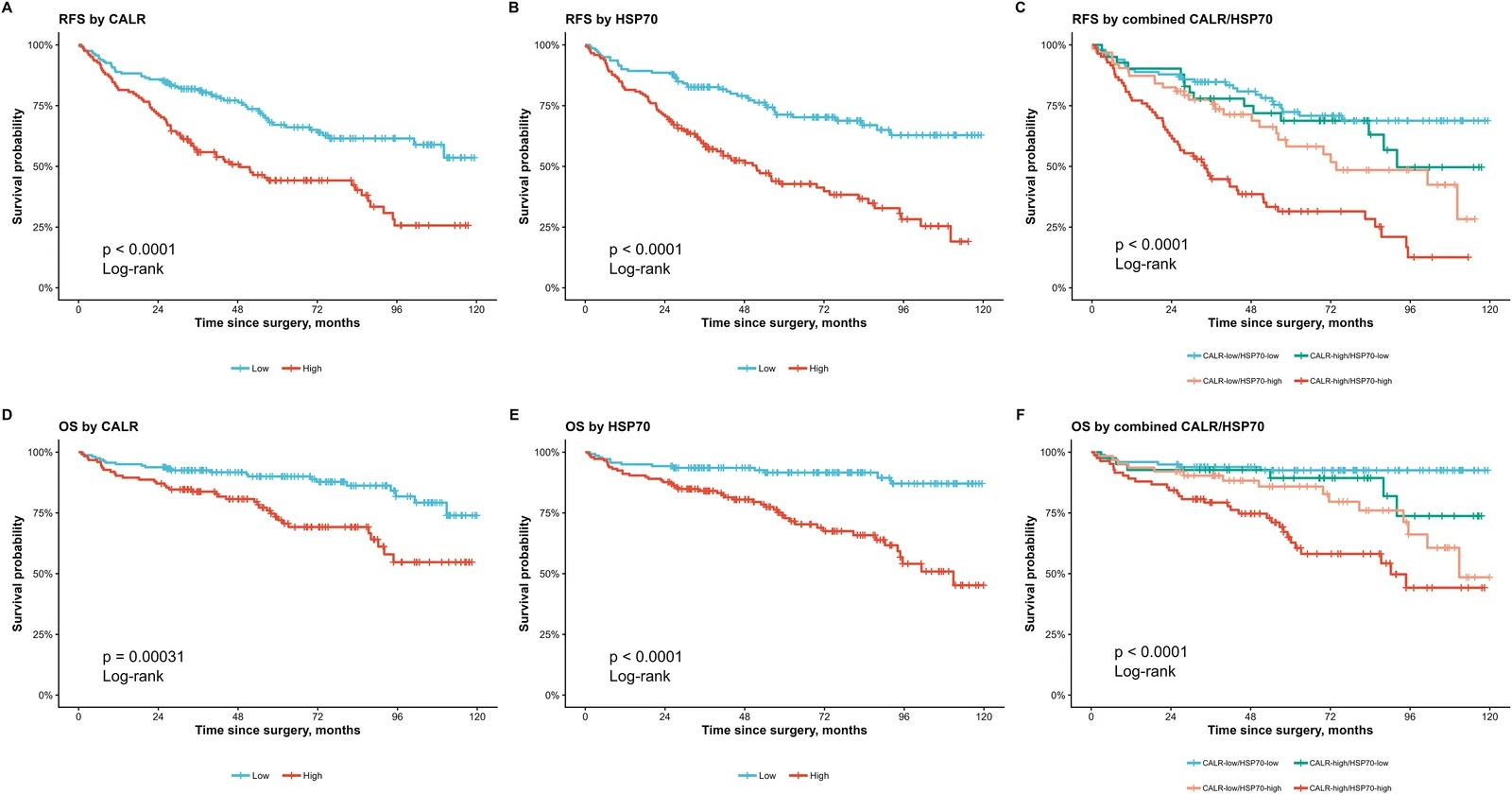
Kaplan-Meier survival curves according to CALR, HSP70, and combined CALR/HSP70 expression groups. Kaplan-Meier curves for recurrence-free survival (RFS) and overall survival (OS) according to CALR expression, HSP70 expression, and combined CALR/HSP70 expression groups. **(A)** RFS according to CALR expression. **(B)** RFS according to HSP70 expression. **(C)** RFS according to combined CALR/HSP70 expression groups. **(D)** OS according to CALR expression. **(E)** OS according to HSP70 expression. **(F)** OS according to combined CALR/HSP70 expression groups. The combined groups were defined as CALR-low/HSP70-low, CALR-high/HSP70-low, CALR-low/HSP70-high, and CALR-high/HSP70-high. P values were derived from the log-rank test, and numbers at risk are provided in **Supplementary Table S10**.

Kaplan-Meier curves for RFS and OS according to CALR and HSP70 expression are shown in **Figure 4**, and numbers at risk are provided in **Supplementary Table S10**. In unadjusted survival analyses, both CALR-high and HSP70-high expression were associated with significantly shorter RFS and OS (all log-rank P values < 0.001).

Consistently, univariable Cox regression analyses demonstrated that CALR high expression was associated with worse RFS (HR 2.22, 95% CI 1.56-3.17, P < 0.001) and OS (HR 2.56, 95% CI 1.51-4.35, P < 0.001), whereas HSP70 high expression was associated with worse RFS (HR 2.70, 95% CI 1.86-3.94, P < 0.001) and OS (HR 4.04, 95% CI 2.18-7.49, P < 0.001) (**Supplementary Table S4**).

After multivariable adjustment including adjuvant treatment (**Table 3**), CALR high expression was no longer independently associated with RFS (adjusted HR 1.45, 95% CI 0.99-2.14, P = 0.059), whereas HSP70 high expression remained associated with poorer RFS (adjusted HR 1.85, 95% CI 1.23-2.79, P = 0.003). In reduced exploratory OS models, CALR high expression was not associated with OS (adjusted HR 1.25, 95% CI 0.69-2.26, P = 0.454), while HSP70 high expression showed a directionally consistent association with poorer OS (adjusted HR 2.27, 95% CI 1.15-4.49, P = 0.018). Because there were 59 deaths, OS multivariable estimates were interpreted cautiously. Sensitivity analyses excluding adjuvant treatment and stratified Cox analyses for proportional-hazards diagnostics are summarized in **Supplementary Table S8**.

**Table 3:** Multivariable Cox regression analyses of CALR and HSP70 expression for recurrence-free survival and overall survival.

Endpoint	CALR, adjusted HR (95% CI)	*P* value	HSP70, adjusted HR (95% CI)	*P* value
RFS	1.45 (0.99-2.14)	0.059	1.85 (1.23-2.79)	0.003
OS, exploratory	1.25 (0.69-2.26)	0.454	2.27 (1.15-4.49)	0.018

High expression was compared with low expression as the reference category. RFS models were adjusted for age, histology, grade, deep myometrial invasion, LVSI, lymph node metastasis, preoperative CA125, and adjuvant treatment. OS models were reduced because of the limited number of deaths and were adjusted for age, non-endometrioid histology, grade 3 disease, advanced clinicopathological stage, LVSI, and adjuvant treatment; OS estimates should be interpreted as exploratory.

Histology-oriented sensitivity analyses are summarized in **Supplementary Table S12**. Non-endometrioid tumors accounted for 41 of 286 cases, including serous carcinoma (n = 24), clear-cell carcinoma (n = 9), and mixed carcinoma (n = 8); therefore, histology was evaluated as endometrioid versus non-endometrioid rather than as separate subtype-specific strata. In full-cohort RFS models including histology group, HSP70-high expression remained associated with poorer RFS (HR 1.96, 95% CI 1.31-2.94; P = 0.001), and the CALR-high/HSP70-high phenotype remained associated with poorer RFS (HR 2.54, 95% CI 1.51-4.25; P < 0.001). In endometrioid-only sensitivity analyses, the corresponding associations remained directionally consistent for HSP70-high expression (HR 2.49, 95% CI 1.63-3.82; P < 0.001) and the CALR-high/HSP70-high phenotype (HR 3.55, 95% CI 2.03-6.20; P < 0.001). There was no clear evidence of interaction by histology group for RFS, although these analyses were limited by the small non-endometrioid subgroup.

### 3.6 Prognostic value of combined CALR/HSP70 expression groups

The prognostic relevance of combined CALR/HSP70 expression groups was further examined (**Figure 4**; **Table 4**; **Supplementary Tables S5**, **S8**, **S10**). In univariable analyses, compared with the CALR-low/HSP70-low group, the CALR-high/HSP70-high group had the worst prognosis, with significantly increased risks of RFS events (HR 4.19, 95% CI 2.62-6.69, P < 0.001) and death (HR 7.01, 95% CI 3.07-15.97, P < 0.001). The CALR-low/HSP70-high group was also associated with inferior RFS (HR 1.91, 95% CI 1.11-3.28, P = 0.019) and OS (HR 3.62, 95% CI 1.47-8.87, P = 0.005), whereas the CALR-high/HSP70-low group was not significantly associated with either endpoint.

**Table 4:** Multivariable Cox regression analyses of combined CALR/HSP70 expression groups for recurrence-free survival and overall survival.

Endpoint	Group (n/events)	Adjusted HR (95% CI)	P value
RFS	CALR-high/HSP70-low (41/15)	1.14 (0.60-2.18)	0.684
RFS	CALR-low/HSP70-high (63/27)	1.58 (0.91-2.77)	0.107
RFS	CALR-high/HSP70-high (83/58)	2.30 (1.36-3.88)	0.002
OS, exploratory	CALR-high/HSP70-low (41/6)	1.50 (0.50-4.55)	0.472
OS, exploratory	CALR-low/HSP70-high (63/15)	2.68 (1.05-6.86)	0.040
OS, exploratory	CALR-high/HSP70-high (83/31)	2.77 (1.09-6.99)	0.031

The CALR-low/HSP70-low group was used as the reference category. For the reference group, n/events were 99/26 for RFS and 99/7 for OS. RFS multivariable models were adjusted for age, histology, grade, deep myometrial invasion, LVSI, lymph node metastasis, preoperative CA125, and adjuvant treatment. OS models were reduced because of the limited number of deaths and were adjusted for age, non-endometrioid histology, grade 3 disease, advanced clinicopathological stage, LVSI, and adjuvant treatment; OS estimates should be interpreted as exploratory. FDR-adjusted P values for OS combined-group comparisons are presented in **Supplementary Table S8**.

In multivariable Cox regression analyses including adjuvant treatment (**Table 4**), the CALR-high/HSP70-high group remained associated with poorer RFS (adjusted HR 2.30, 95% CI 1.36-3.88, P = 0.002). The CALR-low/HSP70-high and CALR-high/HSP70-low groups were not independently associated with RFS after adjustment. In exploratory OS models, the CALR-low/HSP70-high and CALR-high/HSP70-high groups showed nominal associations with poorer OS, but these estimates did not remain below the FDR threshold and should be interpreted cautiously because of the limited number of deaths. No independent association was observed for the CALR-high/HSP70-low group with either endpoint.

### 3.7 Predictive performance of the clinical and integrated models

**Figure 5:**
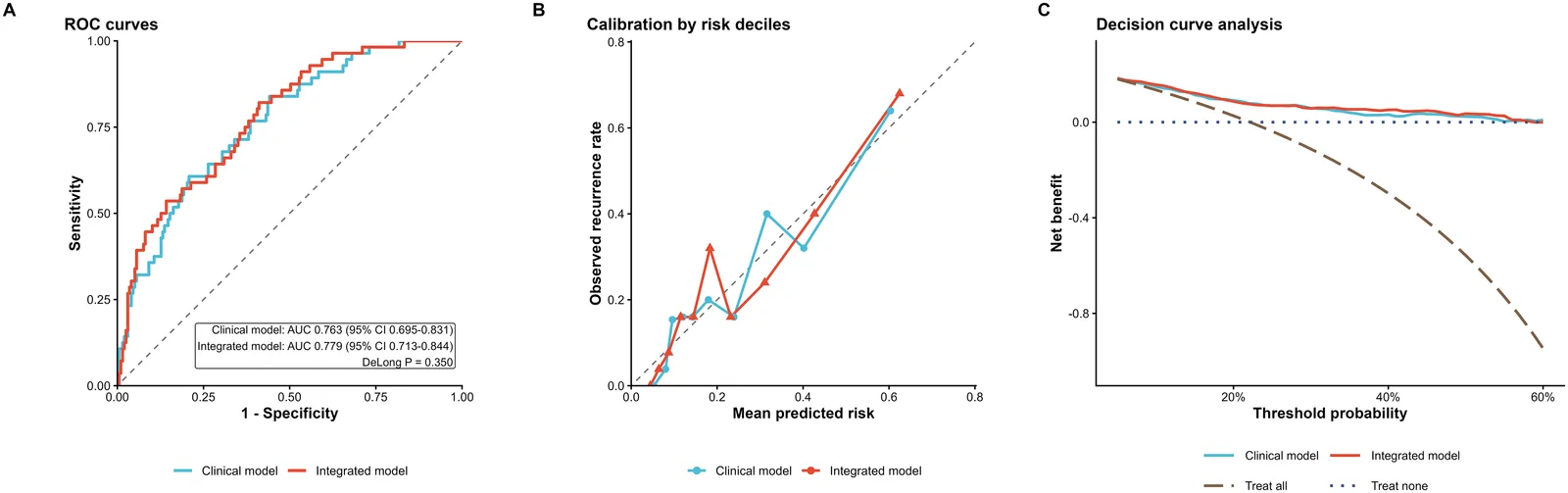
Performance of the clinical and integrated models for 3-year recurrence risk prediction. **(A)** Receiver operating characteristic curves of the clinical model and the integrated model, with apparent AUCs and 95% confidence intervals. **(B)** Calibration curves of the clinical model and the integrated model. **(C)** Decision curve analysis of the clinical model and the integrated model. The clinical model included age, histology, grade, deep myometrial invasion, LVSI, lymph node metastasis, preoperative CA125, and adjuvant treatment. The integrated model additionally included CALR expression, HSP70 expression, CD8 density, and CD68 density. Bootstrap optimism-corrected model performance is summarized in **Table 5**.

The performance of the clinical model and the integrated model for 3-year recurrence risk prediction is summarized in **Table 5**; **Figure 5**. Of the 286 patients, 253 were eligible for the 3-year recurrence analysis, including 56 recurrence events and 197 non-events; 33 recurrence-free patients with less than 36 months of follow-up were excluded from this analysis. The clinical model yielded an apparent AUC of 0.763 (95% CI 0.695-0.831), whereas the integrated model yielded an apparent AUC of 0.779 (95% CI 0.713-0.844). The apparent AUC difference was not statistically significant (DeLong P = 0.350). After bootstrap optimism correction, the clinical and integrated models had similar AUCs (0.720 [95% CI 0.648-0.786] and 0.717 [95% CI 0.651-0.785], respectively). Additional model-performance summaries are provided in **Supplementary Table S9**.

**Table 5:** Comparison of the clinical and integrated models for 3-year recurrence risk prediction.

Model	Estimate type	AUC (95% CI)	Brier score	Calibration intercept/slope	Sensitivity/specificity
Clinical model	Apparent	0.763 (0.695-0.831)	0.145	0.000 / 1.000	0.607 / 0.792
Integrated model	Apparent	0.779 (0.713-0.844)	0.142	0.000 / 1.000	0.821 / 0.589
Clinical model	Optimism-corrected	0.720 (0.648-0.786)	0.159	-0.004 / 0.797	0.554 / 0.756
Integrated model	Optimism-corrected	0.717 (0.651-0.785)	0.161	0.007 / 0.731	0.755 / 0.538

The clinical model included age, histology, grade, deep myometrial invasion, LVSI, lymph node metastasis, preoperative CA125, and adjuvant treatment. The integrated model additionally included CALR expression, HSP70 expression, CD8 density, and CD68 density. The 3-year recurrence model included 253 patients with 56 recurrence events and 197 non-events. Apparent AUCs were compared using the DeLong test (P = 0.350). Optimism-corrected estimates were obtained using 1,000 bootstrap resamples. Additional calibration metrics are presented in **Supplementary Table S9**.

The integrated model showed a small apparent increase in AUC and sensitivity, but this did not translate into validated incremental performance after internal validation. The optimism-corrected Brier score was similar for the clinical and integrated models (0.159 vs. 0.161), and the optimism-corrected calibration slope was lower for the integrated model (0.731) than for the clinical model (0.797). Calibration plots and decision curve analysis suggested no clear clinically meaningful advantage of the integrated model over the clinical model across the examined threshold range.

## 4 Discussion

In this study, we evaluated two immunogenic cell death-related markers, CALR and HSP70, in a surgically treated endometrial cancer cohort and found several consistent patterns. First, both markers showed clear intertumoral heterogeneity on immunohistochemistry with high inter-observer reproducibility. Second, high CALR and high HSP70 expression were each associated with adverse clinicopathological features, including grade 3 disease, deep myometrial invasion, LVSI, lymph node metastasis, and advanced clinicopathological stage. Third, tumors with high CALR or HSP70 expression showed lower CD8+ cell density and higher CD68+ cell density. Fourth, HSP70 retained a more consistent association with RFS than CALR, whereas OS analyses were exploratory because of the limited number of deaths. Finally, the combined CALR/HSP70 grouping, especially the CALR-high/HSP70-high phenotype, provided clearer RFS separation than either marker alone, while the integrated prediction model did not show a validated improvement over the clinical model after bootstrap optimism correction.

These findings are biologically plausible, but they also need to be interpreted in the context of the dual and sometimes opposing roles of CALR and HSP70 in cancer biology. Both proteins are endoplasmic reticulum stress-related chaperones and are widely discussed as damage-associated molecular patterns in immunogenic cell death (21). When translocated to the cell surface or released extracellularly, CALR and HSP70 can contribute to phagocyte recruitment, antigen uptake, and downstream T-cell priming. At the same time, however, tissue overexpression detected by conventional immunohistochemistry does not necessarily reflect productive immunogenic signaling (22). In particular, high intracellular HSP70 is well known to stabilize proteostasis, blunt apoptosis, and support tumor cell survival under stress, while the biological meaning of CALR appears strongly context-dependent and may differ according to cellular compartment, treatment exposure, and tumor type (15, 23).

The HSP70-related findings in our cohort are broadly in line with the older endometrial cancer literature. Nanbu and colleagues reported that HSP70 expression in endometrial carcinoma was associated with non-endometrioid histology, poor differentiation, p53 positivity, and loss of sex steroid receptors, suggesting that HSP70 marks a biologically aggressive subset (17). In a subsequent study from the same group, HSP70-positive tumors were linked to poorer survival, although the marker did not remain independently significant in that smaller series (18). Experimental work has also shown that suppressing HSP70 in endometrial cancer cells can reduce invasion and enhance radiosensitivity (24). Against that background, our observation that HSP70 retained a robust association with RFS, with directionally consistent but exploratory OS findings, appears credible and may indicate that, in routine pathology material, HSP70 is a stronger readout of persistent malignant stress adaptation than CALR.

The CALR results deserve a more careful discussion because they do not fully mirror the limited endometrial cancer-specific literature. Xu et al. reported that low CALR expression was associated with aggressive progression and poor prognosis in endometrial cancer, particularly in a doxorubicin-related treatment context (16). Our data point in the opposite direction, with high CALR expression associating with aggressive pathology and contributing to adverse outcomes in univariable analysis. This discrepancy should not be dismissed as trivial. CALR biology is known to be highly context-dependent, and the prognostic implications of total intracellular CALR differ from those of membrane-exposed CALR. In addition, cohort composition, histologic distribution, treatment setting, scoring thresholds, and whether the analysis captures baseline biology or therapy-related stress responses may all influence the observed direction of association (12, 25). A recent systematic review across multiple carcinomas also suggested that high CALR expression is frequently linked to nodal metastatic behavior, supporting the possibility that in some epithelial tumors CALR upregulation tracks with invasive capacity rather than effective antitumor immunity (25). In other words, our data may be detecting tumor-cell stress adaptation and invasive biology more than canonical ecto-CALR-mediated immunogenicity.

One of the more interesting aspects of the present study is the link between CALR/HSP70 expression and the immune microenvironment. In our cohort, high CALR and high HSP70 expression were each associated with lower CD8+ infiltration and higher CD68+ infiltration. This pattern is more compatible with an immune-suppressed or macrophage-enriched microenvironment than with an efficiently primed antitumor immune response. Prior endometrial cancer studies support this interpretation. A meta-analysis by Guo et al. found that higher intratumoral CD8+ T-cell density was associated with more favorable outcomes in endometrial cancer, while higher tumor-associated macrophage density was linked to worse progression-free survival (9). More recent pathology-based studies have likewise shown that intraepithelial CD8+ lymphocytes are associated with better prognosis in endometrioid endometrial carcinoma (8, 26). On the macrophage side, Soeda et al. demonstrated that tumor-associated macrophages correlated with vascular space invasion and myometrial invasion and were associated with worse survival when enriched at the tumor margin (10). Contemporary reviews further suggest that macrophages in endometrial cancer frequently skew toward a pro-tumor, M2-like state (27). Mechanistically, sustained tumor-cell stress signaling may promote chemokine release, antigen-processing changes, phagocyte recruitment, and macrophage polarization, thereby linking CALR/HSP70 overexpression to a less cytotoxic and more macrophage-enriched immune context. However, our interaction models did not show strong evidence of multiplicative CALR-HSP70 interaction, suggesting that these associations may reflect parallel or additive stress-immune processes rather than a demonstrable synergistic mechanism. Taken together, our immune findings strengthen the argument that CALR/HSP70 overexpression in this setting may sit within a broader program of immune evasion and tumor-promoting inflammation rather than effective immunogenic clearance. At the same time, because we used CD68 as a pan-macrophage marker, we cannot determine whether the observed increase reflects M1-like, M2-like, or mixed macrophage populations (10, 27).

The survival analyses add another layer to this interpretation. CALR was associated with poorer RFS and OS in univariable models, but its effect diminished after accounting for established clinicopathological covariates. HSP70, by contrast, showed a more stable association with RFS, while OS results were interpreted as exploratory. This difference suggests that CALR may overlap more strongly with conventional high-risk pathological features, whereas HSP70 may carry additional information that is not fully captured by grade, invasion depth, LVSI, nodal status, or adjuvant therapy. The combined expression analysis is especially informative here. Patients in the CALR-high/HSP70-high group had the clearest excess RFS risk, while the CALR-high/HSP70-low group was not independently adverse. That pattern argues against a simple additive interpretation and instead suggests that concurrent activation of both markers may identify tumors in which stress adaptation, immune distortion, and invasive potential converge (14, 28). From a pathology standpoint, that is clinically attractive because a two-marker panel remains relatively inexpensive and accessible.

The model analysis should be interpreted with similar restraint. Adding CALR, HSP70, and immune infiltration variables to the clinical model produced a small apparent AUC increase, but the difference was not statistically significant and was not retained after bootstrap optimism correction. This is important. Biomarkers do not automatically become clinically useful simply because they are statistically associated with outcome. In our data, the combined pathology-immune signature may be biologically informative, but it did not provide a validated improvement in discrimination or overall prediction error (29, 30). That does not negate its value; rather, it suggests that these markers are better viewed as pragmatic adjuncts to standard clinicopathological assessment than as standalone prognostic tools.

Several limitations should be acknowledged. First, this was a retrospective single-center study, and the number of outcome events, particularly deaths, was limited for multivariable OS modeling; therefore, OS estimates should be interpreted as exploratory. Second, the biomarker assessment was based on semiquantitative immunohistochemistry rather than digital whole-slide quantification or compartment-specific assays, although inter-observer reproducibility was high. Third, the retrospective source data recorded immune infiltration as averaged cells per high-power field rather than field-level raw counts or fully spatially annotated immune compartments. Fourth, our analysis measured overall tumor-cell expression rather than membrane-exposed CALR or extracellular HSP70, which are closer to the canonical immunogenic cell death framework. Fifth, the immune analysis was intentionally simple and did not distinguish macrophage polarization states, broader lymphocyte subsets, or spatial immune compartments. Sixth, we did not integrate TCGA-style molecular classification. This is increasingly important in modern endometrial cancer stratification, where POLE mutation status, mismatch repair deficiency, p53 abnormality, and NSMP biology substantially shape prognosis and treatment implications (6, 7). Likewise, LVSI continues to carry prognostic significance even within molecularly defined subgroups. Future work should therefore test CALR and HSP70 within molecularly annotated cohorts and determine whether their value is concentrated in specific subtypes rather than in endometrial cancer as a whole. The absence of molecular classification is particularly important in the present study because MMR-deficient and POLE-mutated endometrial cancers may have distinct immune phenotypes. Accordingly, the present data cannot determine whether CALR/HSP70-high tumors represent a stress-immune pattern independent of molecular subtype or partly overlap with MMRd or POLE-mutated tumors. CALR and HSP70 should therefore be interpreted as accessible pathology-based stress-immune markers and not as substitutes for MMRd, POLE-mutated, p53-abnormal, or NSMP molecular classification.

Despite these limitations, the study has several practical strengths. It focuses on markers that are technically feasible in routine pathology laboratories, captures both clinicopathological and immune dimensions, and examines not only single-marker effects but also a biologically sensible combined-expression phenotype. Within the current molecular-classification landscape, these markers may add pathology-accessible information on tumor-cell stress adaptation and broad cytotoxic-versus-myeloid immune remodeling, but their incremental value requires validation in molecularly annotated cohorts.

Overall, our findings support a model in which CALR and HSP70 overexpression in endometrial cancer is linked less to successful antitumor immunogenicity and more to aggressive tumor behavior, macrophage-rich immune remodeling, and inferior RFS. Among the two markers, HSP70 appears to have more stable prognostic relevance, whereas the CALR/HSP70 double-high phenotype provides the clearest high-risk RFS signal. Because OS and prediction-model analyses showed important statistical limitations, these findings should be viewed as hypothesis-generating and require validation in larger, molecularly characterized cohorts, including MMRd, POLE-mutated, p53-abnormal, and NSMP subgroups.

## Data Availability

The original contributions presented in the study are included in the article/**Supplementary Material**. Further inquiries can be directed to the corresponding author.
