# Supplementary material for: Calreticulin and heat shock protein 70 expression, immune infiltration, and prognostic stratification in endometrial cancer: a retrospective cohort study
Source: Front Oncol. 2026 Aug 17;16:1884530. doi: 10.3389/fonc.2026.1884530 (PMC13526587; doi:10.3389/fonc.2026.1884530)
Supplement: Supplementary file 1 [file Table1.docx]

**Supplementary Tables**

**Supplementary Table S1. Clinicopathological comparison between CALR-high and CALR-low groups.**

| **Characteristic** | **Low  N = 162** | **High  N = 124** | **Overall  N = 286** | ***P*-value^1^** |
| --- | --- | --- | --- | --- |
| Age, years, Median (Q1, Q3) | 56.0 (50.0, 62.0) | 58.0 (52.0, 63.5) | 57.0 (51.0, 63.0) | 0.12 |
| BMI, kg/m², Median (Q1, Q3) | 27.5 (24.3, 31.0) | 27.7 (23.9, 31.0) | 27.6 (24.1, 31.0) | 0.75 |
| Menopausal status, n (%) |  |  |  | 0.55 |
| Pre-menopause | 14 (8.6%) | 14 (11%) | 28 (9.8%) |  |
| Post-menopause | 148 (91%) | 110 (89%) | 258 (90%) |  |
| Hypertension, n (%) | 37 (23%) | 27 (22%) | 64 (22%) | 0.89 |
| Diabetes, n (%) | 24 (15%) | 26 (21%) | 50 (17%) | 0.21 |
| Preoperative CA125, U/mL, Median (Q1, Q3) | 20.8 (14.3, 27.9) | 17.6 (13.0, 24.4) | 19.8 (13.7, 27.2) | 0.021 |
| Histology, n (%) |  |  |  | 0.96 |
| Endometrioid | 140 (86%) | 105 (85%) | 245 (86%) |  |
| Serous | 13 (8.0%) | 11 (8.9%) | 24 (8.4%) |  |
| Clear cell | 5 (3.1%) | 4 (3.2%) | 9 (3.1%) |  |
| Mixed | 4 (2.5%) | 4 (3.2%) | 8 (2.8%) |  |
| Histological grade, n (%) |  |  |  | <0.001 |
| G1 | 65 (40%) | 39 (31%) | 104 (36%) |  |
| G2 | 66 (41%) | 35 (28%) | 101 (35%) |  |
| G3 | 31 (19%) | 50 (40%) | 81 (28%) |  |
| Tumor size, cm, Median (Q1, Q3) | 3.2 (2.5, 3.8) | 3.4 (2.6, 4.2) | 3.3 (2.5, 3.9) | 0.14 |
| Myometrial invasion, n (%) |  |  |  | <0.001 |
| <50% | 127 (78%) | 70 (56%) | 197 (69%) |  |
| ≥50% | 35 (22%) | 54 (44%) | 89 (31%) |  |
| Cervical stromal involvement, n (%) | 13 (8.0%) | 29 (23%) | 42 (15%) | <0.001 |
| LVSI, n (%) | 37 (23%) | 57 (46%) | 94 (33%) | <0.001 |
| Serosa/adnexa involvement, n (%) | 8 (4.9%) | 22 (18%) | 30 (10%) | <0.001 |
| Lymph node metastasis, n (%) | 13 (8.0%) | 33 (27%) | 46 (16%) | <0.001 |
| FIGO stage, n (%) |  |  |  | <0.001 |
| I | 127 (78%) | 60 (48%) | 187 (65%) |  |
| II | 12 (7.4%) | 11 (8.9%) | 23 (8.0%) |  |
| III | 19 (12%) | 39 (31%) | 58 (20%) |  |
| IV | 4 (2.5%) | 14 (11%) | 18 (6.3%) |  |
| Adjuvant treatment, n (%) |  |  |  | <0.001 |
| None | 91 (56%) | 39 (31%) | 130 (45%) |  |
| Radiotherapy | 34 (21%) | 24 (19%) | 58 (20%) |  |
| Chemotherapy | 20 (12%) | 27 (22%) | 47 (16%) |  |
| Chemoradiotherapy | 17 (10%) | 34 (27%) | 51 (18%) |  |

^1^Wilcoxon rank sum test; Fisher's exact test

Continuous variables are presented as median (Q1, Q3), and categorical variables are presented as n (%). *P* values were calculated using the Wilcoxon rank-sum test for continuous variables and Fisher’s exact test for categorical variables. Percentages may not sum to 100% due to rounding

**Supplementary Table S2. Clinicopathological comparison between HSP70-high and HSP70-low groups.**

| **Characteristic** | **Low  N = 140** | **High  N = 146** | **Overall  N = 286** | **p-value^1^** |
| --- | --- | --- | --- | --- |
| Age, years, Median (Q1, Q3) | 56.0 (50.0, 63.0) | 58.0 (52.0, 63.0) | 57.0 (51.0, 63.0) | 0.26 |
| BMI, kg/m², Median (Q1, Q3) | 27.7 (24.5, 30.6) | 27.1 (23.8, 31.1) | 27.6 (24.1, 31.0) | 0.90 |
| Menopausal status, n (%) |  |  |  | 0.55 |
| Pre-menopause | 12 (8.6%) | 16 (11%) | 28 (9.8%) |  |
| Post-menopause | 128 (91%) | 130 (89%) | 258 (90%) |  |
| Hypertension, n (%) | 31 (22%) | 33 (23%) | 64 (22%) | >0.99 |
| Diabetes, n (%) | 25 (18%) | 25 (17%) | 50 (17%) | 0.88 |
| Preoperative CA125, U/mL, Median (Q1, Q3) | 20.7 (14.2, 30.2) | 18.1 (13.2, 25.1) | 19.8 (13.7, 27.2) | 0.052 |
| Histology, n (%) |  |  |  | 0.010 |
| Endometrioid | 128 (91%) | 117 (80%) | 245 (86%) |  |
| Serous | 6 (4.3%) | 18 (12%) | 24 (8.4%) |  |
| Clear cell | 5 (3.6%) | 4 (2.7%) | 9 (3.1%) |  |
| Mixed | 1 (0.7%) | 7 (4.8%) | 8 (2.8%) |  |
| Histological grade, n (%) |  |  |  | 0.005 |
| G1 | 61 (44%) | 43 (29%) | 104 (36%) |  |
| G2 | 51 (36%) | 50 (34%) | 101 (35%) |  |
| G3 | 28 (20%) | 53 (36%) | 81 (28%) |  |
| Tumor size, cm, Median (Q1, Q3) | 3.3 (2.5, 3.9) | 3.3 (2.5, 3.9) | 3.3 (2.5, 3.9) | 0.70 |
| Myometrial invasion, n (%) |  |  |  | 0.007 |
| <50% | 107 (76%) | 90 (62%) | 197 (69%) |  |
| ≥50% | 33 (24%) | 56 (38%) | 89 (31%) |  |
| Cervical stromal involvement, n (%) | 11 (7.9%) | 31 (21%) | 42 (15%) | 0.001 |
| LVSI, n (%) | 32 (23%) | 62 (42%) | 94 (33%) | <0.001 |
| Serosa/adnexa involvement, n (%) | 8 (5.7%) | 22 (15%) | 30 (10%) | 0.012 |
| Lymph node metastasis, n (%) | 9 (6.4%) | 37 (25%) | 46 (16%) | <0.001 |
| FIGO stage, n (%) |  |  |  | <0.001 |
| I | 112 (80%) | 75 (51%) | 187 (65%) |  |
| II | 9 (6.4%) | 14 (9.6%) | 23 (8.0%) |  |
| III | 16 (11%) | 42 (29%) | 58 (20%) |  |
| IV | 3 (2.1%) | 15 (10%) | 18 (6.3%) |  |
| Adjuvant treatment, n (%) |  |  |  | <0.001 |
| None | 87 (62%) | 43 (29%) | 130 (45%) |  |
| Radiotherapy | 21 (15%) | 37 (25%) | 58 (20%) |  |
| Chemotherapy | 17 (12%) | 30 (21%) | 47 (16%) |  |
| Chemoradiotherapy | 15 (11%) | 36 (25%) | 51 (18%) |  |

^1^Wilcoxon rank sum test; Fisher's exact test

Continuous variables are presented as median (Q1, Q3), and categorical variables are presented as n (%). P values were calculated using the Wilcoxon rank-sum test for continuous variables and Fisher’s exact test for categorical variables. Percentages may not sum to 100% due to rounding.

**Supplementary Table S3. Univariable logistic regression analyses of CALR and HSP70 high expression for adverse pathological features.**

| **Outcome** | **CALR, OR (95% CI)** | ***P* value** | **HSP70, OR (95% CI)** | ***P* value** |
| --- | --- | --- | --- | --- |
| Grade 3 | 2.86 (1.69–4.89) | <0.001 | 2.28 (1.35–3.93) | 0.002 |
| Deep myometrial invasion | 2.80 (1.68–4.72) | <0.001 | 2.02 (1.21–3.40) | 0.007 |
| LVSI | 2.87 (1.74–4.81) | <0.001 | 2.49 (1.50–4.20) | <0.001 |
| Lymph node metastasis | 4.16 (2.12–8.57) | <0.001 | 4.94 (2.38–11.32) | <0.001 |
| Advanced clinicopathological stage | 4.51 (2.59–8.07) | <0.001 | 4.08 (2.30–7.49) | <0.001 |
| Aggressive pathology composite | 3.59 (2.13–6.18) | <0.001 | 3.17 (1.93–5.29) | <0.001 |

High expression was compared with low expression as the reference category. Advanced clinicopathological stage was defined as stage III-IV based on available clinicopathological staging information. The adverse pathology summary endpoint was defined according to the prespecified criteria described in the Methods section.

**Supplementary Table S4. Univariable Cox regression analyses of CALR and HSP70 expression for RFS and OS.**

| **Endpoint** | **CALR, HR (95% CI)** | ***P* value** | **HSP70,HR (95% CI)** | ***P* value** |
| --- | --- | --- | --- | --- |
| RFS | 2.22 (1.56–3.17) | <0.001 | 2.70 (1.86–3.94) | <0.001 |
| OS | 2.56 (1.51–4.35) | <0.001 | 4.04 (2.18–7.49) | <0.001 |

High expression was compared with low expression as the reference category.

**Supplementary Table S5. Univariable Cox regression analyses of combined CALR/HSP70 expression groups for RFS and OS.**

| **Endpoint** | **Comparison group (reference: CALR-low/HSP70-low)** | **HR (95% CI)** | ***P* value** |
| --- | --- | --- | --- |
| RFS | CALR-high/HSP70-low vs CALR-low/HSP70-low | 1.39 (0.74–2.62) | 0.310 |
| RFS | CALR-low/HSP70-high vs CALR-low/HSP70-low | 1.91 (1.11–3.28) | 0.019 |
| RFS | CALR-high/HSP70-high vs CALR-low/HSP70-low | 4.19 (2.62–6.69) | <0.001 |
| OS | CALR-high/HSP70-low vs CALR-low/HSP70-low | 2.11 (0.71–6.28) | 0.180 |
| OS | CALR-low/HSP70-high vs CALR-low/HSP70-low | 3.62 (1.47–8.87) | 0.005 |
| OS | CALR-high/HSP70-high vs CALR-low/HSP70-low | 7.01 (3.07–15.97) | <0.001 |

**Notes:** The CALR-low/HSP70-low group was used as the reference category.

**Supplementary Table S6. Inter-observer reproducibility of immunohistochemical assessment for CALR and HSP70.**

| **Marker** | **N** | **Total-score ICC (95% CI)** | **High/low agreement** | **Cohen's kappa (95% CI)** | **Interpretation** |
| --- | --- | --- | --- | --- | --- |
| CALR | 286 | 0.949 (0.934-0.962) | 90.9% | 0.817 (0.747-0.881) | Excellent total-score reproducibility and strong high/low classification agreement |
| HSP70 | 286 | 0.936 (0.921-0.950) | 91.3% | 0.825 (0.756-0.887) | Excellent total-score reproducibility and strong high/low classification agreement |

ICC was calculated for the semiquantitative total IHC score. Cohen's kappa and high/low agreement were calculated for dichotomized expression status using the prespecified score threshold of 0-5 versus 6-12. CI, confidence interval; ICC, intraclass correlation coefficient; IHC, immunohistochemistry.

**Supplementary Table S7. Sensitivity analyses for IHC score handling, multiplicity correction, and immune-pattern interpretation.**

| **Analysis** | **Marker or model** | **Summary of results** | **Interpretation** |
| --- | --- | --- | --- |
| Primary high/low threshold (score 6-12 vs. 0-5) | CALR | All five primary adverse pathological features remained FDR-significant; adjusted OR range for primary features: 2.73-4.37. Secondary adverse pathology summary: OR 3.68, P < 0.001. | Supports the primary CALR high/low threshold. |
| Primary high/low threshold (score 6-12 vs. 0-5) | HSP70 | All five primary adverse pathological features remained FDR-significant; adjusted OR range for primary features: 1.92-4.73. Secondary adverse pathology summary: OR 2.94, P < 0.001. | Supports the primary HSP70 high/low threshold, with caution for borderline grade 3 association. |
| Continuous total score per SD | CALR | Associations with all adverse pathology outcomes were directionally consistent and FDR-significant; OR range: 1.53-2.26. | Suggests that dichotomization did not create the overall direction of association. |
| Continuous total score per SD | HSP70 | Associations with all adverse pathology outcomes were directionally consistent and FDR-significant; OR range: 1.57-3.04. | Suggests that dichotomization did not create the overall direction of association. |
| Alternative threshold: score >=5 | CALR/HSP70 | Associations were generally directionally consistent with the primary analysis; most remained FDR-significant. | Supports robustness to a lower high-expression threshold. |
| Alternative threshold: score >=7 | CALR/HSP70 | Associations were generally directionally consistent; HSP70 with deep myometrial invasion was attenuated (OR 1.55, P = 0.101). | Supports overall but not universal robustness; borderline findings were interpreted cautiously. |
| Immune-pattern interaction models | CALR-by-HSP70 interaction | Main effects on CD8 and CD68 density were evident, but formal CALR-by-HSP70 interaction tests did not show strong multiplicative interaction. | Supports descriptive macrophage-skew interpretation rather than a claimed synergistic mechanism. |

FDR adjustment used the Benjamini-Hochberg method within related analysis families. The adverse pathology summary endpoint was treated as a secondary descriptive endpoint because of partial overlap with component pathological features.

**Supplementary Table S8. Survival model sensitivity analyses, events-per-variable assessment, and proportional-hazards diagnostics.**

| **Endpoint/model** | **Exposure** | **n/events** | **EPV** | **Adjusted HR (95% CI)** | **P value** | **Interpretation** |
| --- | --- | --- | --- | --- | --- | --- |
| RFS model with adjuvant treatment | CALR-high vs. low | 286/126 | 14.0 | 1.45 (0.99-2.14) | 0.059 | Not independently significant after adjustment. |
| RFS model with adjuvant treatment | HSP70-high vs. low | 286/126 | 14.0 | 1.85 (1.23-2.79) | 0.003 | Retained RFS association. |
| RFS model without adjuvant treatment | CALR-high vs. low | 286/126 | 15.8 | 1.48 (1.01-2.19) | 0.046 | Directionally consistent sensitivity result. |
| RFS model without adjuvant treatment | HSP70-high vs. low | 286/126 | 15.8 | 2.04 (1.37-3.05) | <0.001 | Directionally consistent sensitivity result. |
| RFS combined model with adjuvant treatment | CALR-high/HSP70-high vs. CALR-low/HSP70-low | 286/126 | 11.5 | 2.30 (1.36-3.88) | 0.002 | Double-high phenotype retained RFS association. |
| RFS stratified Cox sensitivity | CALR-high/HSP70-high vs. CALR-low/HSP70-low | 286/126 | 15.8 | 2.41 (1.43-4.06) | <0.001 | Supports robustness after PH-related stratification. |
| Exploratory OS reduced model with adjuvant treatment | HSP70-high vs. low | 286/59 | 8.4 | 2.27 (1.15-4.49) | 0.018 | Exploratory because EPV <10. |
| Exploratory OS combined reduced model with adjuvant treatment | CALR-high/HSP70-high vs. CALR-low/HSP70-low | 286/59 | 6.6 | 2.77 (1.09-6.99) | 0.031 | Nominal association; FDR-adjusted P = 0.059. |
| Exploratory OS stratified Cox sensitivity | CALR-high/HSP70-high vs. CALR-low/HSP70-low | 286/59 | 11.8 | 2.41 (0.94-6.19) | 0.066 | Directionally consistent but not statistically significant. |

EPV, events per variable; PH, proportional hazards. OS analyses were interpreted as exploratory because of limited deaths. Schoenfeld residual testing did not suggest a clear biomarker-specific PH concern for RFS terms; stratified Cox sensitivity analyses are shown for robustness.

**Supplementary Table S9. Apparent and bootstrap optimism-corrected performance of 3-year recurrence prediction models.**

| **Model** | **Estimate type** | **AUC (95% CI)** | **Brier score** | **Calibration intercept** | **Calibration slope** | **Sensitivity** | **Specificity** |
| --- | --- | --- | --- | --- | --- | --- | --- |
| Clinical model without adjuvant | Apparent | 0.746 (0.672-0.820) | 0.146 | 0.000 | 1.000 | 0.768 | 0.635 |
| Clinical model without adjuvant | Optimism-corrected | 0.708 (0.633-0.782) | 0.159 | 0.011 | 0.823 | 0.727 | 0.595 |
| Integrated model without adjuvant | Apparent | 0.769 (0.698-0.840) | 0.142 | 0.000 | 1.000 | 0.661 | 0.782 |
| Integrated model without adjuvant | Optimism-corrected | 0.712 (0.641-0.782) | 0.160 | 0.008 | 0.747 | 0.596 | 0.741 |
| Clinical model with adjuvant | Apparent | 0.763 (0.695-0.831) | 0.145 | 0.000 | 1.000 | 0.607 | 0.792 |
| Clinical model with adjuvant | Optimism-corrected | 0.720 (0.648-0.786) | 0.159 | -0.004 | 0.797 | 0.554 | 0.756 |
| Integrated model with adjuvant | Apparent | 0.779 (0.713-0.844) | 0.142 | 0.000 | 1.000 | 0.821 | 0.589 |
| Integrated model with adjuvant | Optimism-corrected | 0.717 (0.651-0.785) | 0.161 | 0.007 | 0.731 | 0.755 | 0.538 |

The 3-year recurrence prediction analysis included 253 patients, with 56 recurrence events and 197 non-events. Apparent AUCs were estimated using the DeLong method. Optimism-corrected AUC confidence intervals were estimated using bootstrap percentile intervals from 1,000 resamples.

**Supplementary Table S10. Numbers at risk for Kaplan-Meier survival curves at prespecified time points.**

| **Endpoint** | **Marker** | **Group** | **N** | **0 mo** | **24 mo** | **48 mo** | **72 mo** | **96 mo** | **120 mo** |
| --- | --- | --- | --- | --- | --- | --- | --- | --- | --- |
| RFS | CALR | CALR-low | 162 | 162 | 139 | 89 | 59 | 25 | 0 |
| RFS | CALR | CALR-high | 124 | 124 | 89 | 48 | 27 | 10 | 0 |
| RFS | HSP70 | HSP70-low | 140 | 140 | 124 | 86 | 58 | 24 | 0 |
| RFS | HSP70 | HSP70-high | 146 | 146 | 104 | 51 | 28 | 11 | 0 |
| RFS | Combined | CALR-low/HSP70-low | 99 | 99 | 87 | 61 | 42 | 17 | 0 |
| RFS | Combined | CALR-high/HSP70-low | 41 | 41 | 37 | 25 | 16 | 7 | 0 |
| RFS | Combined | CALR-low/HSP70-high | 63 | 63 | 52 | 28 | 17 | 8 | 0 |
| RFS | Combined | CALR-high/HSP70-high | 83 | 83 | 52 | 23 | 11 | 3 | 0 |
| OS | CALR | CALR-low | 162 | 162 | 152 | 109 | 77 | 34 | 0 |
| OS | CALR | CALR-high | 124 | 124 | 108 | 76 | 42 | 16 | 0 |
| OS | HSP70 | HSP70-low | 140 | 140 | 132 | 100 | 72 | 31 | 0 |
| OS | HSP70 | HSP70-high | 146 | 146 | 128 | 85 | 47 | 19 | 0 |
| OS | Combined | CALR-low/HSP70-low | 99 | 99 | 94 | 71 | 52 | 22 | 0 |
| OS | Combined | CALR-high/HSP70-low | 41 | 41 | 38 | 29 | 20 | 9 | 0 |
| OS | Combined | CALR-low/HSP70-high | 63 | 63 | 58 | 38 | 25 | 12 | 0 |
| OS | Combined | CALR-high/HSP70-high | 83 | 83 | 70 | 47 | 22 | 7 | 0 |

Numbers at risk were calculated from the corresponding time-to-event variables at the indicated time points. RFS was defined as recurrence or death, whichever occurred first.

**Supplementary Table S11. Available screening-level comparison between included and excluded records.**

| **Group** | **n** | **Available date range** | **Surgery performed** | **Complete clinicopathological data** | **CALR IHC complete** | **HSP70 IHC complete** | **Follow-up complete** |
| --- | --- | --- | --- | --- | --- | --- | --- |
| Included | 286 | 2016-01-04 to 2021-05-02 | 286 | 286 | 286 | 286 | 286 |
| Excluded | 126 | 2021-05-29 to 2023-12-20 | 104 | 102 | 108 | 116 | 108 |

The comparison was limited to screening-level variables available for both included and excluded records. Exclusion reasons are detailed in Figure 1. IHC, immunohistochemistry.

**Supplementary Table S12. Histology-oriented sensitivity analyses for CALR/HSP70 expression and survival outcomes.**

| **Analysis** | **Result** | **Interpretation** |
| --- | --- | --- |
| Histology distribution | Endometrioid tumors accounted for 245/286 cases (85.7%); non-endometrioid tumors accounted for 41/286 cases (14.3%), including serous carcinoma (n = 24), clear-cell carcinoma (n = 9), and mixed carcinoma (n = 8). | Separate four-level histology-specific survival models were not used because serous, clear-cell, and mixed carcinoma groups were sparse. |
| CALR expression by histology group | CALR-high expression was observed in 105/245 endometrioid tumors (42.9%) and 19/41 non-endometrioid tumors (46.3%); Fisher exact P = 0.735. | CALR-high expression was not materially imbalanced by endometrioid versus non-endometrioid histology. |
| HSP70 expression by histology group | HSP70-high expression was observed in 117/245 endometrioid tumors (47.8%) and 29/41 non-endometrioid tumors (70.7%); Fisher exact P = 0.007. | HSP70-high expression was more frequent in non-endometrioid tumors, supporting the need to account for histological composition. |
| Double-high phenotype by histology group | The CALR-high/HSP70-high phenotype was observed in 67/245 endometrioid tumors (27.3%) and 16/41 non-endometrioid tumors (39.0%); Fisher exact P = 0.057. | The double-high phenotype was numerically more frequent in non-endometrioid tumors but did not reach conventional statistical significance. |
| Full-cohort RFS model including histology group: CALR-high | HR 1.57 (95% CI 1.06-2.32); P = 0.024. | Directionally consistent RFS association after including endometrioid versus non-endometrioid histology group. |
| Full-cohort RFS model including histology group: HSP70-high | HR 1.96 (95% CI 1.31-2.94); P = 0.001. | HSP70-high expression remained associated with poorer RFS after accounting for histology group. |
| Full-cohort RFS model including histology group: CALR-high/HSP70-high | HR 2.54 (95% CI 1.51-4.25); P < 0.001. | The double-high phenotype remained associated with poorer RFS after accounting for histology group. |
| Endometrioid-only RFS sensitivity model: CALR-high | HR 1.83 (95% CI 1.19-2.83); P = 0.006. | The CALR association was directionally consistent when analysis was restricted to endometrioid tumors. |
| Endometrioid-only RFS sensitivity model: HSP70-high | HR 2.49 (95% CI 1.63-3.82); P < 0.001. | The HSP70 RFS association persisted within endometrioid tumors. |
| Endometrioid-only RFS sensitivity model: CALR-high/HSP70-high | HR 3.55 (95% CI 2.03-6.20); P < 0.001. | The double-high RFS association persisted within endometrioid tumors. |
| Interaction by histology group for RFS | P for interaction was 0.498 for CALR, 0.486 for HSP70, and 0.867 for the combined CALR/HSP70 group. | There was no clear evidence that RFS associations differed by endometrioid versus non-endometrioid histology, although interaction analyses were limited by the small non-endometrioid subgroup. |

RFS, recurrence-free survival; HR, hazard ratio; CI, confidence interval. Histology group was defined as endometrioid versus non-endometrioid. OS analyses are not emphasized here because multivariable OS models were exploratory owing to the limited number of deaths.
